# Primary Mediastinal Ewing sarcoma presenting as a massive lung lesion with a mediastinal shift

**DOI:** 10.1002/ccr3.4857

**Published:** 2021-10-10

**Authors:** Fateen Ata, Mohamad Safwan Aljafar, Areej Marwan Mohammed, Salman Mirza, Abdul Aziz Zafar

**Affiliations:** ^1^ Department of Internal Medicine Hamad Medical Corporation Doha Qatar; ^2^ Department of Radiology Hamad Medical Corporation Doha Qatar; ^3^ Weill Cornell Medicine ‐ Qatar Doha Qatar

**Keywords:** Ewing sarcoma, mediastinum, peripheral primitive neuroectodermal tumor, PPNET, primary pulmonary Ewing sarcoma

## Abstract

Primary pulmonary Ewing sarcoma can present as a massive mass in the left hemithorax covering the entire lung and can press the pulmonary artery and cause a significant mediastinal shift.

## Graphical abstract

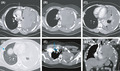

## 1 CLINICAL IMAGE CASE

Ewing sarcoma usually arises from the pelvis, axial skeleton, and femur. Rarely, it may arise from soft tissue in the chest wall. We present a 16‐year‐old male patient with a large mass in the left hemothorax covering the entire lung and pressing the pulmonary artery. Histopathology confirmed the mass as Ewing's sarcoma.

**FIGURE 1:**
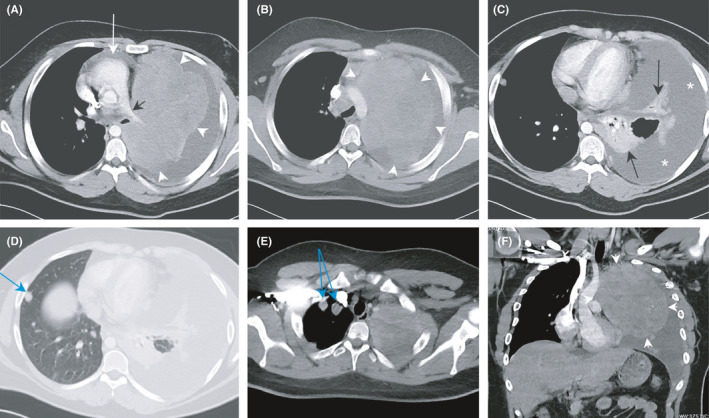
(A and B) Axial images of the contrast‐enhanced CT chest. A mass lesion can be seen in the left hemithorax (between white arrowheads), which is attenuating the left main pulmonary artery (black arrow) and causing a shift of the mediastinum to the right (white arrow). (C) An almost total collapse of the left lung (black arrows) and sizable left pleural effusion (white asterisk). (D and E) Multiple pleural‐based nodular lesions noted in the right hemithorax (blue arrows). (F) Coronal reformat image of the contrast‐enhanced CT chest

A 16‐year‐old male patient presented with a one‐month history of progressively increasing nocturnal cough and dyspnea. Physical examination was normal apart from supplemental oxygen requirement (2 lpm). A chest X‐ray revealed a complete whiteout of the left hemithorax. A CT‐scan revealed an 18‐cm mediastinal mass with pleural effusion, pressing the pulmonary artery and causing a mediastinal shift to the right (Figure 1). A CT‐guided biopsy was done, and histopathology revealed a malignant small round blue cell tumor consistent with Ewing's sarcoma. Positron emission tomography scan confirmed the primary nature of ES with no uptake elsewhere. The patient was planned for chemotherapy, but he traveled abroad.

Although a rare site for peripheral primitive neuroectodermal tumor (PPNET), multiple cases of pulmonary ES are reported to date. Tumor sizes are reported to range between 4 and 9 cm.^1^ Pulmonary ES tends to be aggressive with a poor outcome, with around 50% mortality rate in the reported cases.^1^ The patients with aggressive PES need rapid identification and treatment with surgery and chemotherapy (vincristine, doxorubicin, and ifosfamide), and radiotherapy wherever possible, to reduce mortality. Similar clinical presentation (dyspnea and cough) with a PPNET involving the whole hemithorax has been reported before.^2^ The patient had an aggressive course with death on day 15 of treatment.

## 2 CONCLUSION

Primary pulmonary Ewing Sarcoma can present as a massive lung mass with compressive signs and symptoms. Due to a poor prognosis, rapid identification followed by surgery and chemotherapy can aid in mortality reduction.

## CONFLICTS OF INTEREST

None of the authors have any conflict of interest to disclose.

## AUTHOR CONTRIBUTIONS

FA involved in conceptualization, methodology, literature review, manuscript writing, image selection, and arrangement, review, and revisions in the final manuscript. AM and MA involved in literature review and manuscript writing. SM: involved in radiology part of the image case, figure, and legend provision. AZ: involved in supervision, case identification, review, and revisions in the final manuscript. All authors reviewed and approved the final version of the manuscript.

## ETHICAL APPROVAL

Written consent was taken from the patient for the brief clinical case and accompanying images before submission. The clinical image case was approved by the Medical Research Centre (MRC), Qatar, before submission.

## CONSENT

Written informed consent was obtained from the patient for publication of this case report and accompanying images.

## Data Availability

Not applicable.
